# Bone Mineral Density of the Spine in 11,898 Chinese Infants and Young Children: A Cross-Sectional Study

**DOI:** 10.1371/journal.pone.0082098

**Published:** 2013-12-06

**Authors:** Haiqing Xu, Zhiwei Zhao, Hong Wang, Ming Ding, Aiqin Zhou, Xiaoyan Wang, Ping Zhang, Christopher Duggan, Frank B. Hu

**Affiliations:** 1 Department of Child Health Care, Hubei Maternal and Child Health Hospital, Wuhan, China; 2 Department of Nutrition, Harvard School of Public Health, Boston, Massachusetts, United States of America; 3 Children's Hospital, Boston, Massachusetts, United States of America; Faculté de médecine de Nantes, France

## Abstract

**Background:**

Bone mineral density (BMD) increases progressively during childhood and adolescence and is affected by various genetic and environmental factors. The aim of this study was to establish reference values for lumbar BMD in healthy Chinese infants and young children and investigate its influencing factors.

**Methods and Findings:**

Healthy children aged 0 to 3 years who underwent regular physical examinations at the Child Health Care Clinic of Hubei Maternal and Child Health Hospital (N = 11,898) were recruited for this study. We also chose 379 preterm infants aged 0 to 1 years to preliminarily explore the development of BMD in this special population. BMD (g/cm^2^) measurements of the lumbar spine (L2–L4) were carried out with dual-energy X-ray absorptiometry and a questionnaire was administered to full-term children's parents to gather information on various nutritional and lifestyle factors as well as mothers' nutritional supplement use during pregnancy. Lumbar BMD significantly increased with age among both boys and girls (*p*<0.05), with fastest growth observed during the first postnatal year. There was no significant difference in lumbar BMD between boys and girls of similar age (*p*>0.05), either among healthy reference children or preterm infants. However, BMD values in preterm infants were significantly lower than those in term infants 3 to 8 months old (*p*<0.05) after adjustment for gestational age. Multivariable linear regression analysis indicated significant positive associations between lumbar BMD of healthy children and the child's age and current weight, mother's weight gain during pregnancy, birth weight, children's outdoor activity duration and children's physical activity duration.

**Conclusion:**

Our study provides reference values of lumbar BMD for healthy Chinese children aged 0 to 3 years and identifies several influencing factors.

## Introduction

Bone mass gradually increases during childhood and adolescence, influenced by genetic, nutritional, and environmental factors. Achieving peak bone mineralization during early adulthood has been proven to decrease the risk of osteoporosis [1]. Since the main bone mineral is calcium, BMD can also serve as an indicator of calcium metabolism [2].

Presently, dual-energy X-ray absorptiometry (DXA) is preferentially recommended for the determination of BMD due to its rapid assessment and low radiation dose with high precision and accuracy [3]–[5] compared with other measurements. Several studies have established normal BMD data for children and adolescents, but they were restricted to particular regions and ethnicities and/or were often based on a relatively small sample size [6]–[7]. We therefore carried out a cross-sectional study to establish BMD reference values for the lumbar spine of healthy Chinese children aged 0 to 3 years and to evaluate the influence of age, sex, weight, height, birth weight, children's physical activity and outdoor exercise durations and calcium and vitamin D supplementation on these values. Moreover, because the development of BMD in preterm infants is a growing focus for researchers and clinical practitioners, we also carried out a preliminary study to compare the BMD of preterm infants and healthy reference children during the first year of life.

## Subjects and Methods

### Study Subjects

We recruited11,898 Chinese children ages 0 to 3 years (5306 girls and 6592 boys) from among healthy children attending the Child Health Care Clinic of Hubei Maternal and Child Health Hospital in Wuhan, China. Included were children who had been born full term (gestational age ≥37 weeks). Excluded were children with congenital or metabolic bone disease, as well as children with clinical evidence of chronic renal, hepatic, or gastrointestinal diseases. In addition, children with length or weight <10^th^ or >90^th^ percentile according to National Standard for Urban Children Growth and Development (2005) [8] were excluded. Subjects were divided into 15 groups based on age. Written informed consent was obtained from at least one parent, and the study was approved by the Ethics Committee of Hubei Maternal and Child Health Hospital.

As a comparison group, we selected 379 preterm infants (gestational age 32–36 weeks; 205 boys and 174 girls) and conducted DXA scan of these infants. Excluded were preterm infants with chronic diseases and those prescribed medicines affecting bone structure or metabolism. A corrected gestational age of 40 weeks, that is gestation plus postnatal age, was applied for each preterm infant when determining the age for detection. All preterm infants received vitamin D 400 IU/day after birth.

### Questionnaire Interview

We developed an original questionnaire which was administered to full-term children's parents by trained children health care professionals through person to person interviews. This questionnaire was designed to gather information on birth length and weight, duration of outdoor activities per day by the babies (as a surrogate for duration of exposure to sunlight), duration of physical activity by the babies per day (including playing with toys, sitting, crawling, walking, running, and baby exercise performed with parents), mother's weight gain and calcium supplementation during pregnancy. The questionnaire assessed the quality and frequency of daily consumption of milk and/or breast milk during the last week by children, and information on additional calcium and vitamin D supplementation of term infants was obtained from parents.

### Anthropometric Measurements

Infant length and weight were measured in triplicate before BMD examination. Subjects were weighed while wearing minimal clothing on the digital scale (WS-RT-1D, Wuhan Kangwa Science and Technology Limited Company China) to the nearest 0.01 kg. Length was measured to the nearest 0.1 centimeter with the baby supine on the scale without shoes and to calculate the distance from top head to the heel by using fixed head and moveable foot board simultaneously. Weight for age z-scores and height for age z-scores were calculated using the national child growth standards [8].

### BMD Assessment

BMD (g/cm^2^) measurements of the lumbar spine (L2–L4) were carried out with dual-energy X-ray absorptiometry (435A102, Norland A CooperSurgical Company, USA) with software version 3.8, strictly following the manufacturer's operating instructions. Lumbar vertebrae L2 to L4 were chosen for measurement because previous studies indicate that the trabecular bone is more sensitive to mineral changes than the cortical bone [9]–[10]. All measurements were performed by a trained nurse at the Department of Child Health Care of Hubei Maternal and Child Health Hospital. Quality-control scans were performed daily using a standard spine phantom; precision (coefficient of variation on repeated scan analysis) using the phantom was 0.9%. All scans were performed with the child in a supine position and without sedation. Children were directly observed at all times by the person performing the scan. The scanning procedure was interrupted if any movement artifact was noted, and a repeat scan was performed when the child was pacified. Immobilization or swaddling was applied when necessary.

### Statistical Analysis

The results are expressed as mean ± SD. Two-tailed *t*-tests were used to compare BMD in different age and sex groups, as well as between term and preterm children. Linear and multiple regression analyses (using the backwards elimination method) were used to identify factors associated with BMD. Differences were considered statistically significant at P<0.05. Data were analyzed using SPSS version 13.0 (SPSS Inc., Chicago, IL, USA).

## Results

### Clinical Characteristics of Reference Children

Among 11,898 term infants studied, mean (SD) birth weight and gestational age were 3232 (469) g and 39.6 (1.3) weeks, respectively. Median age at time of DXA was 6.7 months (interquartile range [IQR]: 3.4 to 12.2 months). The mean weight and height were 9.13 (3.331) kg and 70 (11.1) cm, respectively. The mean weight-for-age z scores and length-for-age z scores for the term infants were 0.12 (0.07) and 0.09 (0.06), respectively. Among term infants, 84.6% were either being exclusively or partially breast-fed. Solid food was introduced at a median of 173 days (interquartile range 94 to 162 days). The clinical characteristics of reference children aged 0∼3 years are represented in Table 1.

**Table 1:** Clinical characteristics of 11,898 healthy reference Chinese children aged 0∼3 years.

Variable	Mean ± SD or n (%)
Male sex	6592, (55.4%)
Gestational age at birth, weeks	39.6±1.3
History of Breastfeeding	10,066(84.6%)
Age at the examination, months	9.27±7.60
Weight, kg	9.13±3.33
Weight-for-age Z scores	0.12±0.07
Height, cm	70.0±11.1
Height-for- age Z scores	0.09±0.06
Maternal pregnancy weight gain, kg	16.4±7.8
Maternal pregnancy calcium supplement use	7,590 (63.8%)
Birth weight, kg	3.232±0.469
Birth length, cm	49.6±5.3
Daily milk and/or breast milk intake, mL	750±251
Outdoor activity duration, h/d (in spring or autumn)	4.1±2.3
Physical activity duration, h/d	1.3±0.8
Calcium supplement use	3,926(33.0%)
Vitamin D supplement use	11,303(95.0%)

### Lumbar BMD Differences between Healthy Reference Children and Preterm Infants

**Figure 1:**
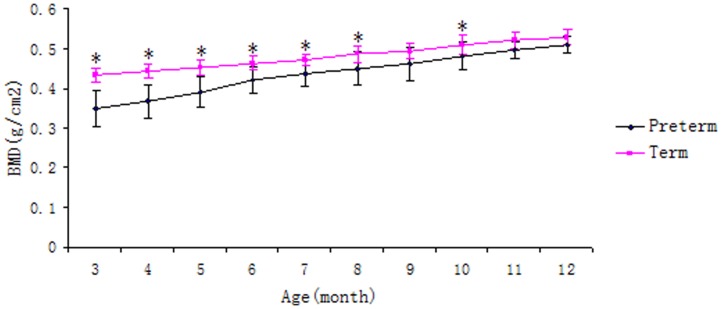
The development of BMD of term and gestational age-adjusted preterm infants aged 3∼12 months (*p<0.05, compared with the preterm of the same age after adjusting for the gestation).

Mean BMD of the lumbar spine in boys and girls, term and gestational age-adjusted preterm, at different ages, are presented in Tables 2 and 3. The BMD of the lumbar spine (L2–L4) for term children increased significantly with age before 3 years among both boys and girls (*p*<0.05), with the most rapid increase during the first year after birth. The same trend was identified for gestational-age-adjusted preterm infants 3 to 12 months of age (Figure 1), and the BMD of gestational-age-adjusted preterm infants was significantly lower than that of term infants in the same age group before 9 month-old (Table 4).

**Table 2:** BMD values of term children aged 0∼3 years (g/cm^2^) (Mean±SD).

Age (Months)	Male		Female		P value	Combined BMD
	N	BMD	N	BMD		
1	516	0.407±0.066	345	0.402±0.068	0.42	0.405±0.067
2	792	0.416±0.045	642	0.415±0.043	0.14	0.415±0.044
3	783	0.433±0.042	615	0.431±0.040	0.13	0.432±0.041
4	369	0.444±0.048	250	0.439±0.047	0.03	0.444±0.047
5	290	0.453±0.047	278	0.449±0.046	0.57	0.452±0.047
6	786	0.464±0.042	604	0.462±0.047	0.90	0.463±0.044
7∼8	401	0.487±0.042	387	0.482±0.042	0.48	0.485±0.042
9∼10	732	0.507±0.037	637	0.504±0.036	0.18	0.506±0.037
10∼12	418	0.517±0.036	361	0.509±0.035	0.30	0.513±0.035
13∼15	375	0.539±0.051	328	0.530±0.041	0.50	0.535±0.047
16∼18	325	0.551±0.054	289	0.547±0.055	0.71	0.549±0.055
19∼21	177	0.564±0.062	145	0.553±0.056	0.84	0.559±0.059
22∼24	250	0.576±0.056	162	0.573±0.062	0.96	0.575±0.059
25∼30	221	0.591±0.055	161	0.584±0.044	0.88	0.588±0.049
31∼36	157	0.608±0.052	102	0.599±0.042	0.19	0.604±0.048

**Table 3:** BMD values of preterm infants at gestation-adjusted age of 3∼12 months (g/cm^2^) (X±SD).

Age (Months)	Male		0	Female		P value	Combined BMD
	N	BMD		N	BMD		
3	27	0.349±0.101		18	0.345±0.068	0.83	0.348±0.113
4	24	0.368±0.092		16	0.360±0.076	0.54	0.366±0.099
5	18	0.392±0.103		21	0.391±0.084	0.45	0.391±0.097
6	28	0.424±0.102		22	0.419±0.096	0.75	0.421±0.082
7	12	0.443±0.095		13	0.433±0.101	0.40	0.438±0.078
8	19	0.452±0.109		16	0.449±0.124	0.41	0.450±0.102
9	21	0.464±0.102		24	0.460±0.099	0.70	0.461±0.102
10	18	0.481±0.087		14	0.478±0.104	0.42	0.480±0.082
11	17	0.498±0.092		12	0.493±0.086	0.89	0.496±0.053
12	21	0.511±0.067		18	0.509±0.056	0.77	0.510±0.051

**Table 4:** Comparison of BMD values between term and gestational-age-adjusted preterm infants of 3∼12 months (X±SD).

Age (Months)	Preterm(N)	Term(N)	T test	P value
3	0.348±0.113(45)	0.432±0.041(3693)	−5.86	<0.001
4	0.366±0.099(40)	0.444±0.047(619)	−4.95	<0.001
5	0.391±0.097(39)	0.452±0.047(568)	−5.06	<0.001
6	0.421±0.082(50)	0.463±0.044(1390)	−2.83	<0.001
7	0.438±0.078(25)	0.472±0.032(394)	−3.21	<0.001
8	0.450±0.102(35)	0.486±0.048(394)	−4.09	<0.001
9	0.461±0.102(45)	0.492±0.045(650)	0.27	0.79
10	0.480±0.082(32)	0.510±0.057(719)	−3.42	0.002
11	0.496±0.053(29)	0.520±0.051(400)	−0.39	0.70
12	0.510±0.051(39)	0.528±0.049(379)	−1.51	0.14

Two sample students't-tests were performed to compare the BMD of lumbar spine (L1–L4) for male and female term and preterm infants in the same age group (Table 2, 3). The results revealed that there was no significant difference in lumbar BMD between boys and girls of similar age (p>0.05), either among healthy reference children or preterm infants.

### Factors Associated with Lumbar BMD in Healthy Reference Children

Univariate linear regression analysis was used to identify factors associated with the lumbar spine BMD of term children (Table 5). The results revealed positive associations of BMD with age, weight, and height at the time of the DXA examination, mother's weight gain during pregnancy, daily milk and or breast milk intake, outdoor activity duration, physical activity duration, and supplementation of calcium and vitamin D. Lumbar spine BMD was inversely associated with both birth weight and mother's calcium supplementation during pregnancy.

**Table 5:** Univariate linear regression analysis of individual factors associated with BMD in healthy term Chinese children ages 0–3 years.

Variables	Parameter values	T test	P value
Sex	−0.0014	−1.03	0.30
Age (months)	0.0073	128.00	<0.0001
Current weight (kilograms)	0.025	173.00	<0.0001
Current height (centimeters)	0.0054	131.27	<0.0001
Maternal pregnancy weight gain (kilograms)	0.0097	6.33	<0.0001
Maternal pregnancy calcium supplement use	0.01	7.32	<0.0001
Birth weight (kilograms)	0.033	13.67	<0.0001
Birth height (centimeters)	−0.00077	−1.10	0.27
Daily milk and/or breast milk intake (milliliters)	−0.00005	−26.49	<0.0001
Outdoor activity duration (hours/day)	0.07	46.92	<0.0001
Physical activity duration (hours/day)	0.066	141.93	<0.0001
Calcium supplement use	0.064	10.25	<0.0001
Vitamin D supplement use	0.013	90.72	<0.0001

(1)Gender: 0 was assigned for Male and 1 for Female. (2) Maternal pregnancy weight gain: 1 was assigned for pregnancy weight gain being less than 10 kg; 2 for 10 to 15 kg of weight gain and 3 for more than 15 kg of weight gain; (3)Pregnancy calcium supplement use: 0 was assigned for No and 1 for Yes

To identify independent predictors of lumbar spine BMD in term infants, a multiple linear regression model with the backward elimination method was applied (Table 6). Age and weight at the time of the DXA assessment, mother's weight gain during pregnancy, birth weight, outdoor activity duration, and physical activity duration done by the child alone or with parents were all significantly associated with increased lumbar spine BMD.

**Table 6:** Multiple linear regression analysis of influencing factors of BMD in healthy children

Variables	Parameter values	T test	P value	Standard regression coefficient
Sex	−0.0032	−1.58	0.1052	−0.2535
Age (months)	0.0027	3.95	0.0001	0.1442
Current weight (kilograms)	0.0049	3.76	0.0018	0.2639
Pregnancy weight gain (kilograms)	0.0065	2.68	0.0032	0.0646
Birth weight (kilograms)	0.0026	1.89	0.0486	0.0354
Outdoor activity duration (hours/day)	0.0068	3.01	0.0020	0.0542
Physical activity duration (hours/day)	0.0129	2.24	0.0156	0.0956

(1)Gender: 0 was assigned for Male and 1 for Female. (2)Pregnancy weight gain: 1 was assigned for pregnancy weight gain being less than 10 kg, 2 for 10 to 15 kg of weight gain, and 3 for more than 15 kg of weight gain.

## Discussion

BMD is known to increase continuously until its peak during childhood and adolescence [11]–[14], and achieving peak levels is considered important for the prevention of osteoporosis and bone fracture in adulthood [14]. Although several previous studies have established normal reference BMD value for young children and adolescence, relatively little is known about BMD development during the first three years of life, and how prematurity affects BMD is also unclear. To the best of our knowledge, this is the first study in a large sample of Chinese healthy children aged 0 to 3 years that explored the normal development of lumbar spine BMD and its determinants. DXA of lumbar vertebra has proven useful in the measurement of BMD in children due to its accuracy, low radiation dose, short scan time, and ease of use without sedation. Prior studies have suggested that peak bone mass and BMD are influenced by age, height, weight, genetic variations, ethnicity, and environmental factors such as nutrition and physical activity. How these factors contribute to the development of BMD under 3 years remain largely unknown.

Our study revealed that the BMD of the lumbar spine increased significantly with age in the first 3 years among healthy term boys and girls, with the steepest increase during the first year. This trend was consistent with physical development of the child during this stage, suggesting rapid development of spinal bone mass. There was no significant difference in BMD between boys and girls of the same age before 3 years, similar to the findings of Nelson et al [15] and Haiqing et al [16], which may due to the absence of the effect of sex hormones or similar modes of nourishment and lifestyle for children 0 through 3 years. Our results indicate a similar trend for BMD in preterm infants aged 3 to 12 months, and our finding of significantly lower BMD each month compared with term infants indicates inadequate bone mineralization in preterm infants. Few studies have been conducted on the BMD of the infants in large samples. Compared to other Asian populations, the BMD values in our study were higher than those in Japanese and Korea populations of the same age and sex [17]–[18], however, the trend that higher BMD was related to older age was the same. The difference in the BMD results might be due to the different DEXA instruments used in different studies and different nutritional status of the children.

To investigate the factors that may influence the BMD of healthy children, we analyzed the potential factors related to nutritional and lifestyle factors as well as mothers' nutritional status during pregnancy. Consistent with our results, weight has been suggested to be a major influencing factor on BMD for healthy boys and girls [17]–[18]. In multiple regression analyses, we found that children's weight was the strongest determinant of spinal BMD in children under 3 years of age. It is possible that the stress of higher weight stimulates the strengthening of the skeleton through greater bone mineralization. Though some other studies have failed to find a significant correlation between weight and BMD in Chinese children [19]–[20], these discrepancies may reflect differences in the children's age and the bone area in which BMD was measured. Milk and other dairy products are rich in calcium and many high-quality proteins and essential micronutrients. Not only can dairy products provide the calcium that is the major component of skeleton, but the vitamin D in milk may facilitate the absorption of calcium [18]. Several previous studies have found that milk intake was closely related to BMD [21]–[22]. However, our results did not reveal a positive correlation between daily milk consumption and spinal BMD. This may be due in part to the inaccurate reporting of the intake of milk and other dairy products.

Nutrition during pregnancy was also positively related to spinal BMD in term infants in our study, consistent with previous findings [23]. Better nutrition during pregnancy can help establish a solid foundation for the progress of BMD post partum.

In the present study, we found no independent effect of vitamin D supplementation on BMD. This may be due to the fact that it is now a routine in China to supplement the diets of infants. However, we found that spinal BMD was positively correlated with duration of children's outdoor activity. Ultraviolet irradiation in sunlight can trigger a series of biological reactions within the human body that lead to the production of functional endogenous 1,25-dihydroxy vitamin D3 [24]–[25]. Taha et al [26] found that a group of ultra-orthodox Jewish children in the US had higher bone fracture rates and lower spinal BMD, which may due to less exposure to sunlight.

Several studies have indicated that exercise influences BMD at different ages [27]–[29]. Consistent with previous studies, we also found a positive association between physical activity duration and lumbar spine BMD. There is a rapid growth in bone mass during infancy and adolescence. Adequate exercise can prompt the increase of bone mass and BMD through several pathways. One is that the local stress loads generated from muscle contraction during exercise can strengthen the activities of osteoblast cells and osteogenesis [30]–[31]. In addition, exercise can increase levels of IGF-1, which is actively involved in bone formation [32]–[35]. Thus, caregivers should provide infants with appropriate amounts and quality of exercise to help enhance BMD.

There are also some limitations about our study. First, we only chose the lumbar spine (L2–L4) for the BMD measurement, which may not be representative of other skeleton, and the relationship of BMD between lumbar spine and total body skeleton among this study population should also be investigated. Second, although longer physical activity duration was found to be correlated with higher lumbar spine BMD values in our study, how the differences in intensity and types of physical activities contribute to the development of lumbar spine BMD needs to be clarified. Finally, although we identified a significantly lower BMD among preterm infants in this study, further research with more detailed classifications of preterm infants and more background information about these infants is needed to confirm our findings.

## Conclusion

Infancy and adolescence are the critical stages for normal accumulation of bone mass and BMD. Through the present study, we have established normal reference values for spinal BMD among healthy Chinese children aged 0 to 3 years, which can be used as a tool for the assessment and follow-up of Chinese children at risk for low-bone mineralization. The main factors that influence the development of BMD in healthy children include children's body weight, maternal weight gain during pregnancy, and sufficient outdoor activity and exercise.
